# Experimental Application of Methods to Compute Solar Irradiance and Cell Temperature of Photovoltaic Modules

**DOI:** 10.3390/s20092490

**Published:** 2020-04-28

**Authors:** Caio Felippe Abe, João Batista Dias, Gilles Notton, Ghjuvan Antone Faggianelli

**Affiliations:** 1 Photovoltaic Solar Energy Laboratory, Unisinos Unversity, São Leopoldo 93022-750, RS, Brazil; abe.caio@gmail.com (C.F.A.); joaobd@unisinos.br (J.B.D.); 2 Centre Georges Peri, University of Corsica, 20000 Ajaccio, France; faggianelli_ga@univ-corse.fr

**Keywords:** estimation of solar irradiance, estimation of PV cell temperature, PV modeling, PV module as a sensor

## Abstract

Solar irradiance and cell temperature are the most significant aspects when assessing the production of a photovoltaic system. To avoid the need of specific sensors for quantifying such parameters, recent literature presents methods to estimate them through electrical measurements, using the photovoltaic module itself as a sensor. This work presents an application of such methods to data recorded using a research platform at University of Corsica, in France. The methods and the platform are briefly presented and the results are shown and discussed in terms of normalized mean absolute errors (nMAE) and root mean square errors (nRMSE) for various irradiance and cell temperature levels. The nMAE (and nRMSE) for solar irradiance are respectively between 3.5% and 3.9% (4.2% and 4.7%). Such errors on computed irradiance are in the same order of magnitude as those found in the literature, with a simple implementation. For cell temperatures estimation, the nMAE and nRMSE were found to be in the range 3.4%–8.2% and 4.3%–10.7%. These results show that using such methods could provide an estimation for the values of irradiance and cell temperature, even if the modules are not new and are not regularly cleaned, but of course not partially shaded.

## 1. Introduction

### 1.1. Problem and State of Art

The knowledge of the solar energy received by a thermal or photovoltaic (PV) solar system is an essential stage to estimate the performance of the plant and to forecast its future production. Solar radiation data must be available today at a relatively short time step in view to optimize the energy management and to size, in the best possible way, the various subsystems included in the solar plant. However, it appears that these measures of solar radiation are not always available and that some areas in the world do not have solar radiation measurements stations [1] or such measurements are available only with a large acquisition time-step (daily data, monthly average values,…), with limited interest for research. The reasons of this scarcity of solar data are the high investments and maintenance costs of the solar radiation measurement devices, especially for nonprofit institutions, such as schools or universities [2,3]. In some applications, such as automotive ones, several solar measuring devices must be used simultaneously [4], increasing the cost problem. In the case of small-scale PV arrays, such as rooftop PV systems, the much higher costs of pyranometers leads to use of other devices for monitoring solar irradiance [5,6]. The solar irradiance that a crop receives is measured because it affects its biological processes and several measurement devices are necessary, thus, using cheap but reliable irradiance sensors decreases the cost of the experiment [7]. Similarly, for large-scale MW-size PV systems with a large area of PV modules, solar irradiance can be different from one part of the array to other, and several sensors are required for a precise estimation of the solar energy received, increasing the overall cost of the system [8].

Having less expensive solar measuring devices allows the extension of the number of meteorological stations measuring solar irradiance through the world, which are too few in number (not more than one thousand) and not very precise [9,10]. Solar data measured in an area of 30 km around the solar plant can be considered as being usable for a sizing or production estimation but even for these applications, 98% of the stations are too far apart to give accurate information [11]. 

Today, an important topic in solar energy research is the forecasting of the intermittent solar irradiance, which complicates the management of such electrical production. The knowledge of future production of PV plants allows the energy supplier to optimize the energy management, in order to maximize the renewable energy penetration into a power system through e.g., economic dispatch, reserve allocation, and electricity network. An efficient, reliable, and precise measure of solar irradiance is absolutely necessary for good forecasting, as shown in [12], and the possibility to have efficient and cheap solar irradiance sensors is crucial [13].

Various measuring devices can be used for solar irradiance: pyranometers (thermopile-based instruments) or calibrated PV cells, which present different responses according to spectral, angular, and temperature effects [5,14,15,16]. A pyranometer based on a thermal effect has a high time constant and then a relatively long response time, while a PV cell has a quasi-instantaneous response and is significantly cheaper [17]. Some original and interesting new solar radiation measuring methods have been developed, such as the approach used by Oulcaid et al. [18], which employs a low-cost fixed standard camera observing the PV array images and deduces solar irradiance from the variation of colors intensity; the accuracy of this method was calculated using the root mean square error, which was found in the range of 18 to 32 W·m^−^².

One solution in view to reduce the instrumentation cost and to increase the availability of the global solar data, consists of using PV plants in production state of all sizes to estimate the incident solar radiation with a short time granularity, also determining the ambient temperature, which is the second more influencing parameter on PV plant performances.

It is well known that there is a relation more or less complex between the short-circuit current *I_sc_* and the solar irradiance *G* [19]; more information will be provided in the second part of this introduction. Recently, the short-circuit current *I_SC_* of different-type PV modules was described based on the environmental factors under various solar irradiance levels [20,21]. This property can obviously be used to develop irradiance sensors, but it is important to keep in mind the influence of the solar spectrum and the average photon energy (APE) on the relation between *I_sc_* and the solar irradiance *G* (more IR at low irradiance levels, more UV-visible at high irradiance levels) [22], with some differences according to the technology. Five small-scale PV arrays were modeled using solar irradiance, which was measured respectively by a pyranometer and by two PV modules in short-circuit conditions (one CdTe module and one CIS module) [5]; the results have shown a slight overestimation with a normalized root mean square error, nRMSE, equal to 6%–8% of the irradiance measured by the PV modules (a better accuracy for the CdTe module is noted); however, the costs are around 20 times lower. Orsetti et al. [23] developed an inexpensive solar irradiance measurement consisting of 45 × 45 mm PV cells coupled in series and directly connected with a shunt resistor and to a digital sensor interface. Several studies evaluated and compared the performances of different solar irradiance sensors [7,24,25,26] to low cost ones [19,27,28].

A state-of-the-art review [18] on the estimation of solar irradiance from the measurement performed by PV array showed that:

1. An iterative method allows the estimation of the global horizontal irradiance using Perez transposition models and power measurements; an nRMSE of 15.1% was obtained in the best case [29]; another iterative method coupling two combined algorithms has been also used [6]; it was underlined that such iterative approaches can have some convergence problems [30].
2. An approach based on an artificial neural network (ANN) to calculate the solar irradiance from the cell temperature and electrical measurements was developed by Mancilla-David et al. [27]; and ANN methods are efficient but are generally not repeatable because the training phase is based on historic data, which strongly depends on the analyzed system.
3. A closed-form analytical estimator (CFAE) allows the determination of the solar irradiance with an nRMSE between 1.5% and 3.2% [28].
4. A reparameterization of the I-V curve results in a convergent solar irradiance estimator with an nRMSE around 0.87%—for stable irradiance—and 6.65% for a perturbed one [31].
5. An extended Kalman filter has been adopted [8] and presented less accurate results than an analytical model [28] and an immersion and invariance (I&I) model [31] used for the same objective.
6. An overview of methods using temperature and DC electrical measurements (short-circuit, under-load, and open-circuit states) was realized by Vigni et al. [26]; this synthesis presented an nRMSE equal to 6.4%, 6.8%, and 11.3%, respectively, obtained for short-circuit, under-load, and open-circuit states.

Da Costa et al. [32] proposed an irradiance and temperature estimator only using the short-circuit current, the open-circuit voltage *V_oc_*, and operating current and voltage for PV module using a mathematical model and numerical simulations. This method presents a maximum normalized mean bias error, nMBE, of 2.47% in the irradiance and of 2.64% in the temperature.

Moshksar and Ghanbari [33] developed a reliable, yet somewhat complex method to estimate the solar irradiance and PV temperature in the maximum power point (MPP) conditions; the accuracy in terms of normalized absolute relative error, nMAE, is 1.08% (maximum 4.22%) and 0.53% (maximum 0.69%) for respectively the solar irradiance G and the PV cell module temperature *T_c_*. 

Some remarks can be made:

1. All the methods regarding the use of *V_oc_* to compute *T_c_* and the use of *I_sc_* to compute *G* require the PV modules to be disconnected from the inverter to allow such measurements at the extreme points of the I-V curve to be carried out; thus, if a PV power plant is used to realize such a measure, it must be disconnected from the electrical grid and stop its electrical production.
2. For the papers in which a method for computing both *G* and *T_c_* based on the current *I_mpp_* and the voltage *V_mpp_* in maximum power point conditions is used and that can be applied to systems that are under operation [33], it appears that the methods are often more elaborate and involve the determination of several parameters that have to be estimated, but when a computer is employed to collect measurements, no matter how complex the algorithm is, the calculations are easily and rapidly realized.

Our objective is to apply two methods [34,35], which are briefly described in the following section, that are able to measure the solar irradiance and the PV cell temperature of a PV system. They are easy to implement; [34] depends on *I_mpp_* and *V_mpp_*, thus does not require interruption of the electrical production, whereas [35] can be used for modules which are not operating, since it is based on *V_oc_* measurements. 

### 1.2. Measure of the Solar Irradiance and Temperature

In [19,27,28], the operating point of the sensor PV modules is positioned close to the short-circuit *I_sc_*, whose value is proportional to *G*. The sensor PV module could be eliminated if the PV array itself was used as an irradiance sensor. This is possible as long as the PV modules are clean and free of any malfunctioning. However, shifting the operating point would impact the energy production, since at the short-circuit, the power supplied by the module is zero. The inverter is capable of establishing the operating point of the PV module at the maximum power point, in which the current is *I_mpp_* and the voltage is *V_mpp_*. The calculation of *G* using the *I_mpp_* value is described in [34], in which I-V curves under different levels of *G* and *T_c_* were compared, and the effect of *T_c_* on *I_mpp_*, which is very slight, was neglected. Therefore, the changes on *I_mpp_* are considered a function of *G* only. Considering that the relation *I_sc_* / *I_mpp_* is constant, the short-circuit current can be estimated by

(1) $$I_{\mathrm{sc}}\;\approx \;I_{\mathrm{mpp}\;}\;\frac{I_{\mathrm{sc},\mathrm{STC}}}{I_{\mathrm{mpp},\mathrm{STC}\;}}\;,$$

therefore, dividing (1) by $I_{\mathrm{sc},\mathrm{STC}\;}$ provides a reference for the irradiance [34], STC being the standard test conditions ($T_{c}=25\;{}^{\circ}C,\;G=1000$ W·m^−^² and an air mass 1.5 (AM1.5) spectrum. Simplifying (1) thus provides

(2) $$G\;\approx 1000\frac{I_{\mathrm{mpp}}}{I_{\mathrm{mpp},\mathrm{STC}\;}}\;.$$

In [34], such a method to estimate the irradiance presented the relative absolute error smaller than 5% during tests using I-V curves data from 500 to 1000 W·m^−^² and error smaller than 3% using data from a research platform, with the module connected to a microinverter, at around 800 W·m^−^². 

The cell temperature of photovoltaic (PV) modules is also of great significance, since critical parameters depend on it as open-circuit *V_oc_* or maximum power point *V_mpp_* voltages, influencing the PV module production. Regarding both performance testing and operation monitoring, the quantification of cell temperature (*T_c_*) is crucial for assessing the PV device behavior. Temperature measurements performed by means of temperature sensors attached to the back of PV modules present drawbacks, such as the fact that the temperature gradient along the module surface is not considered, since the measurements are punctual [36,37]. In addition to that, the actual cell temperature does not equal the temperature of the rear surface of a module, due to the drop along the different materials that compose the module. Temperature quantification methods that compute *T_c_* as a function of the voltage take into account the temperature of each cell, since the latter are connected in series, thus providing a measure of the average temperature of the module, avoiding the aforementioned drawbacks [36]. Recent literature present methods to compute the cell temperature based on measurements at the output of PV modules. For instance, [35] proposes an application of the translation method presented in [38] to compute the average temperature of PV modules, straightforwardly, from the open-circuit voltage (*V_oc_*) and solar irradiance (*G*), using Equation (3). Such a method can be applied outdoors, when the module is not operating.

(3) $$V_{oc}=V_{oc,STC}\left(1+\beta \;\left(T_{c}-T_{c,STC}\right)\right)\left(1+\delta \left(T_{c}\right)\;\ln\frac{G}{G_{STC}}\right)$$

where $V_{oc,STC}$ is the open-circuit voltage under the standard test condition (STC), where $G_{STC}\;$ = 1000 W·m^−2^ and $T_{c,STC}$ = 25 °C. In addition to that, β is the temperature coefficient of *V_oc_* in 1/°C and $\delta \left(T_{c}\right)$ is given by Equation (4).

(4) $$\delta \left(T_{c}\right)=MT_{c}+N$$

where

(5) $$M=\frac{\delta_{NOCT}-\delta_{LIC}}{T_{c,NOCT}-T_{c,LIC}}\;\;\;\;\;\mathrm{and}\;\;\;\;N=\delta_{NOCT}-MT_{c,NOCT}$$

with

(6) $$\delta_{NOCT}=\frac{1}{ln\left(\frac{G_{NOCT}}{1000}\right)}\left(\frac{V_{oc,NOCT}}{V_{oc,STC}\left(1+\beta \left(T_{c,NOCT}-T_{c,STC}\right)\right)}-1\right)$$

and

(7) $$\delta_{LIC}=\frac{1}{ln\left(\frac{G_{LIC}}{1000}\right)}\left(\frac{V_{oc,LIC}}{V_{oc,STC}\left(1+\beta \left(T_{c,LIC}-T_{c,STC}\right)\right)}-1\right)$$

The abbreviation NOCT refers to nominal operating cell temperature, which presents typical values from 43 to 47 °C for crystalline modules. The test condition for the determination of NOCT is usually called NOCT condition, in which $G_{NOCT}$ = 800 W·m^−2^ and an ambient temperature equal to 20 °C, with a wind speed of 1 m·s^−1^. In turn, LIC stands for low irradiance condition, where $G_{LIC}$ = 200 W·m^−2^ and $T_{c,LIC}$ = 25 °C. It is worth noting that $V_{oc,NOCT}$ and $V_{oc,LIC}$ refer to the open-circuit voltage under NOCT and LIC conditions, respectively. Considering that the datasheets of PV modules hardly present information under LIC condition, usually *M* = 0 and *N* = $\delta_{NOCT}$ in Equations (4) and (5); therefore, Equation (3) can be explicitly solved for *T_c_*, as shown in (8).

(8) $$T_{c}=\left(\frac{V_{oc}}{V_{oc,STC}\left(1+\delta_{NOCT}\;ln\frac{G}{G_{STC}}\right)\;}-1\right)\frac{1}{\beta }+T_{c,STC}$$

In turn, a method introduced in [36] can be applied to PV systems in operation, since the maximum power point (*P_mpp_*) coordinates of voltage (*V_mpp_*) and current (*I_mpp_*), which are established by the inverter, are used to compute *G* and *T_c_*. This way, the operating point of the PV array does not need to be shifted, whereas the use of dedicated temperature and irradiance sensors is avoided, since the PV module acts as the sensor for *G* and *T_c_*. The cell temperature can be computed using

(9) $$T_{C}=\left(\frac{P_{\mathrm{mpp}}\;G_{\mathrm{STC}}}{G\;I_{\mathrm{mpp},\mathrm{STC}}\;V_{\mathrm{mpp}}\left(G\right)\;}-1\right)\frac{1}{\tau \;\gamma }+T_{C,\mathrm{STC}},$$

where γ is the temperature correction factor for the *P_mpp_*. The voltage at the maximum power point is written as a function of *G* given by (10)

(10) $$V_{\mathrm{mpp}}\left(G\right)=V_{\mathrm{mpp},\mathrm{STC}}+V_{\mathrm{oc},\mathrm{STC}\;}\psi \ln\left(\frac{G}{G_{\mathrm{STC}}}\right),$$

with

(11) $$\psi =\frac{V_{\mathrm{mpp},\mathrm{NOCT}}-V_{\mathrm{oc},\mathrm{STC}\;}\;\beta \left(T_{NOCT}-T_{\mathrm{STC}}\right)-V_{\mathrm{mpp},\mathrm{STC}}}{V_{\mathrm{oc},\mathrm{STC}\;}\ln\left(\frac{G_{\mathrm{NOCT}}}{G_{\mathrm{STC}}}\right)}\;.$$

The adjustment factor τ in Equation (9) can be defined for a value so that Equation (9) returns *T_c_* = *T_c,NOCT_*, whereas using *P_mpp_* = *P_mpp,NOCT_* and *G* = *G_NOCT_*, as long as ψ has been adequately computed. 

The studies [34,35] considered simulation and experiments limited to few points. The present study focuses on the application of such methods on experimental cases considering a much larger data amount, aiming to determine the relative mean absolute error (nMAE) and root mean square error (nRMSE) associated with each method. The nMAE is computed using Equation (12), whereas the nRMSE is computed by means of Equation (13). In such equations, $x_{c,i}$ is the ith computed value, $x_{m,i}\;$ is the ith measured value and $\bar{x_{m}}$ is the average of the measured values, and *n* is the number of data.

(12) $$\mathrm{nMAE}=\frac{\sum_{i=1}^{n}\left|x_{c,i}-x_{m,i}\right|}{n\;\bar{x_{m}}}$$

(13) $$\mathrm{nRMSE}=\sqrt{\frac{\sum_{i=1}^{n}{\left(x_{c,i}{}_{i}-x_{m,i}\right)}^{2}}{n}}\;\frac{1}{\bar{x_{m}}}$$

This paper presents the equations regarding methods [34,35], as well as data referring to the PV modules and the measurement system of the DURASOL platform [39], from which a large dataset has been obtained. Such data contain electrical measurements of the modules, as well as the corresponding measurements of *G* and *T_c_*. The methods have been used in conjunction with the electrical measurements to compute *G* and *T_c_*, and their performance has been assessed for each module and method and the results are discussed. 

## 2. Materials and Methods

This section presents the procedures for the experimental application of methods [34,35], applied to the DURASOL data, referring to four 245 W Tenesol PV modules.

### 2.1. The DURASOL Project at University of Corsica

**Figure 1:**
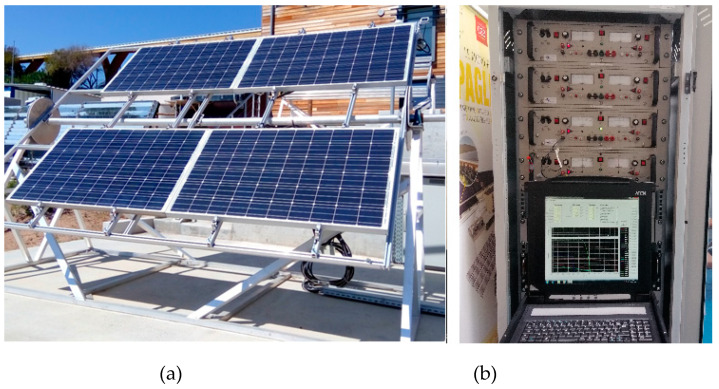
The four Tenesol modules (**a**) and the acquisition and curve display system (**b**).

The experimental data used in this work has been obtained from one of the DURASOL project sites, which is located at the SPE Laboratory UMR CNRS, at University of Corsica, in France. Because the aim of such a project is to support studies on PV module aging [39], data regarding new and old modules are available. The dataset consists of electric measurements on four Tenesol TE245-60M+ modules, along with the corresponding measurements of solar irradiance and module temperature. The data collection covered almost 16 months, during which *V_oc_*, *V_mpp_*, *I_mpp_*, and short-circuit current (*I_sc_*) of each module have been measured every 5 mins. According to [39], each I-V curve consists of 100 points obtained in about 1 s. It is worth mentioning that the irradiance measurements were carried out close to the modules and at the same inclination, using a Kipp & Zonen CMP10 pyranometer. In turn, the temperature was measured by means of PT100 (RTD) attached to the back of each of the PV modules. Figure 1 illustrates the experimental PV system. The modules are installed in a coastal environment subject to salt spray, and there is no cleaning regime. 

Before inclusion to DURASOL platform, two of these modules had been in use for five years, composing a PV array and presenting normal operation. Therefore, these modules are regarded as old. The other two modules are from the same batch; however, had never been exposed to solar radiation before the experiment. They are referred to as new. These two new modules are identified in the present work as A and B, whereas the two old modules are referred as C and D. 

For the application of methods [34,35], some parameters of the module under study must be known under STC and NOCT. Such parameters are organized in Table 1 and have been obtained from flash tests using a solar simulator. The tests were carried out by the manufacturer; therefore, the data refer to the modules as they were new. 

The data in Table 1 are specific for each module and do not present significant deviation when compared to the information provided on the datasheet of the TE245-60M module. Such datasheet information is presented in Table 2, along with α, β, and γ, which are the temperature coefficients for *I_sc_*, *V_oc_*, and *P_mpp_*, respectively. The module has 60 monocrystalline cells, and the NOCT is 45 °C.

It is worth recalling that data under NOCT condition are required in order to apply methods [34,35]. Therefore, the DURASOL experimental data have been filtered, selecting the measurement points under *G* = 800 W·m^−2^ and *T_c_* = 45 °C, with +/− 1% tolerance. The average values of the remarkable I-V curve points are presented in Table 3. 

### 2.2. Using the Methods with DURASOL Data to Compute *T_c_*

For the application of the method described in [35] to compute *T_c_* as a function of the open-circuit voltage of the PV module, the parameter $\delta_{NOCT}$ has to be quantified for each module using Equation (6). Similarly, to employ the method described in [34] to calculate *T_c_* as a function of the voltage at the maximum power point, the parameters ψ and τ must be defined for each case, which has been carried out from Equations (11) and (9) written for the NOCT case. It is worth mentioning that the parameters referring to each module, used in Equations (6), (9), and (11), are those presented in Table 1, Table 2 and Table 3. The calculated parameters are organized in Table 4, for each module. 

## 3. Results and Discussion

This section presents the results of the application of methods described in [34,35] to the DURASOL platform measured data. The first analysis considers the correlation of measured and computed values of *G* and *T_c_*. Further, the absolute mean errors and root mean square errors are presented, allowing us to relate the error magnitude with the corresponding levels of *G* and *T_c_*. Finally, graphical analysis illustrates the behavior of the measured and computed parameters, as well as the associated errors, under steady and transient conditions. 

### 3.1. Correlation between Measured and Computed Parameters

The plots presenting the correlations of measured and computed parameters are shown with reduced number of points, to allow better visualization (i.e., plotted data regarding module D will not totally cover the data previously plotted, related to the other modules). The selection criteria for the removal of points is sequential: one point has been plotted and the following 99 have been skipped, applying such a procedure until the end of the dataset. However, the nMAE and nRMSE values were calculated on the totality of the available data.

**Figure 2:**
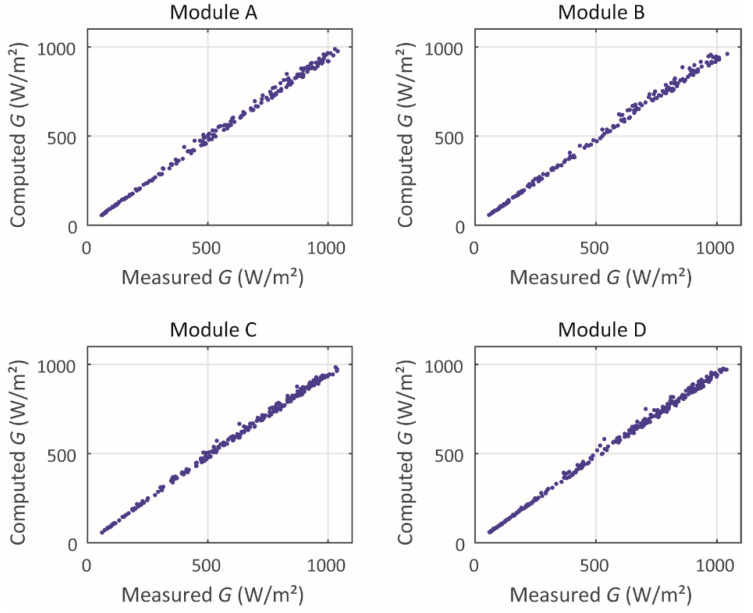
Irradiance correlation.

For the four modules studied, the measured values of irradiance, as well as the values computed based on the *I_mpp_*, by means of [34], are related in Figure 2. The plots in Figure 2 contain points measured from about 50 to 1100 W·m^−2^. The irradiance values under 50 W·m^−^² were not taken into account because generally these values are measured during sunset and sunrise periods and the solar irradiance sensors can be impacted by a mask effect (at high zenith angle).

It appears that the reliability of the irradiance correlation is good because the points are very little scattered around the straight line *Y* = *X*. This good accordance between measured and computed values will be proved by the reliability metrics presented below, in Table 5.

**Figure 3:**
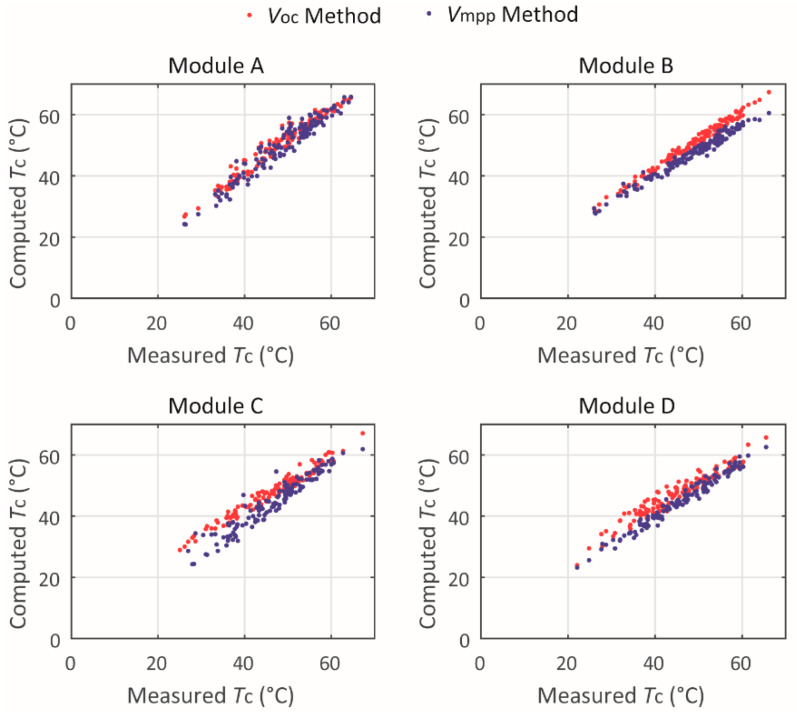
Temperature correlation by open-circuit voltage *V_oc_* method [35] and maximum power point *V_mpp_* method [34].

Correlations between measured and computed values of *T_c_*, per module are illustrated in Figure 3 for *T_c_* calculated by means of the *V_oc_* method and the *V_mpp_* method. It is worth recalling that “*V_oc_* method” refers to [35], whereas “*V_mpp_* method” refers to [34]. 

The points in Figure 3 present some difference with regard to the computed *T_c_*, depending on the method used. Moreover, some points presented quite high dispersion. These issues are discussed further in the paper. It should be mentioned that the data density in Figure 2 and Figure 3 have been reduced by 100 times; however, the errors presented in Table 5, Table 6, Table 7 and Table 8 consider the full dataset.

### 3.2. Mean Absolute Errors and Root Mean Square Errors

The normalized mean absolute errors (nMAE) and the normalized root mean square error (nRMSE) on *G* and *T_c_* have been computed separately per irradiance range and are organized in Table 5, Table 6 and Table 7. Regarding *T_c_*, two tables have been produced: one considering method [35] (Table 6) and the other considering the application of [34] (Table 7). It is worth recalling the scope of use of such methods to determine *T_c_*: the method outlined in [35] considers the use of open-circuit condition, where external measurement of *G* is needed. This method is suitable for determining *T_c_* in situations where a module is outdoors and not in use with an MPPT device. In turn, [34] considers that the module is in use, thus the *P_mpp_* coordinates (*V_mpp_* and *I_mpp_*) are used: *G* is computed from *I_mpp_*, whereas *T_c_* is from *V_mpp_*. Therefore, the operational condition of the system must not be changed to allow computing *G* and *T_c_*, in contrast to other methods presented in literature, which are based on *I_sc_* and *V_oc_* measurements. 

The error levels—both from nMAE and nRMSE—present quite stable behavior for the different irradiance ranges considered. Because the method to compute *G* from the *I_mpp_* described in [36] does not take the effect of temperature into account, the slightly higher error levels for the 600–1000 W·m^−2^ range are not surprising, since higher temperature levels (higher than 25 °C used as reference) are generally reached.

**Table 6:** Normalized nMAE and nRMSE on computed *T_c_* via [35], per irradiance range, in %.

	50–300 W·m^−2^		300–600 W·m^−2^		600–1100 W·m^−2^		50–1100 W·m^−2^	
Module	nMAE	nRMSE	nMAE	nRMSE	nMAE	nRMSE	nMAE	nRMSE
**A**	5.4	6.2	3.2	4.1	5.2	6.2	4.7	5.8
**B**	6.8	7.2	6.8	7.2	5.3	5.7	6.0	6.5
**C**	21.0	21.3	12.5	13.2	4.7	6.1	8.1	10.7
**D**	30.5	30.8	15.7	16.3	4.5	5.4	7.7	9.6

**Table 7:** Normalized nMAE and nRMSE on computed *T_c_* via [34], per irradiance range, in %.

	50–300 W·m^−2^		300–600 W·m^−2^		600–1100 W·m^−2^		50–1100 W·m^−2^	
Module	nMAE	nRMSE	nMAE	nRMSE	nMAE	nRMSE	nMAE	nRMSE
**A**	11.8	12.7	6.9	7.7	3.7	4.7	4.9	6.0
**B**	5.7	6.7	3.2	4.0	4.1	5.0	3.8	4.7
**C**	12.2	12.5	11.0	11.6	6.4	7.4	8.2	9.4
**D**	9.1	9.9	2.8	3.5	3.6	4.4	3.4	4.3

Concerning Table 6 and Table 7, under the first two ranges of irradiance levels, higher nMAE and nRMSE have been found for modules C and D, which are older than modules A and B. Such higher errors are more pronounced when the *V_oc_* method [35] is used. Under the range 600–1100 W/m², it is not possible to establish a relation between the error level and the module condition (new or old). The proposed models (as with many others in literature) require knowledge of the modules parameters; indeed if one considers the parameters referring to the modules "as new", and the modules have suffered aging, then actually the parameters are not known and thus, the modeling results could present higher errors. 

These higher errors for old modules highlight a limitation of our model for the temperature estimation. If adequate aging modeling is incorporated to the current methods, the correction could be carried out based on years of use. Of course, further research is required to establish how such a proposal could be implemented and its feasibility. This is a motivation for further study and improvement of the methods, which could consider PV module aging effects (and their behavior according to the irradiance level) and corresponding measures to correct the models, aiming to reduce the corresponding errors.

Comparing Table 6 and Table 7, it appears that globally the *V_oc_* method is better than the *V_mpp_* method, but it depends on the module and on the solar irradiance range; consequently, it is difficult to conclude on the best method.

The accuracy of the method is higher for the solar irradiance estimation than for the temperature one; in fact, the thermal effect due mainly to the solar irradiance is not instantaneous, i.e., the temperature does not react immediately to a variation of the solar irradiance due to the inertia of the PV module. If the data were averaged on a larger time step, the influence of the module inertia should be reduced and a better accuracy should be obtained. 

**Figure 4:**
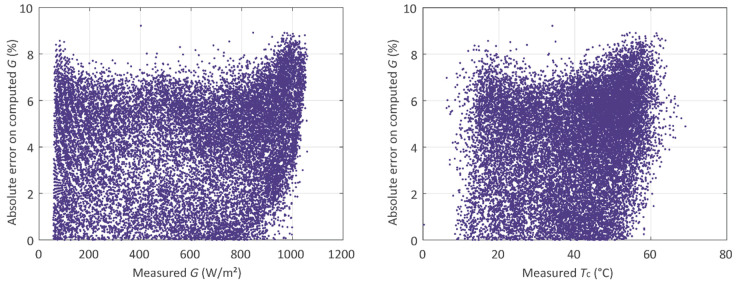
Absolute errors on computed solar irradiance *G* (from *I_mpp_*) and corresponding measured values of *G* and *T*_c._

The errors on *G* and *T_c_* of individual measurements, per irradiance and per cell temperature, are illustrated in Figure 4. 

The errors on *G* computed from the *I_mpp_*, illustrated in Figure 4, present relatively high values for high temperature levels, which is in agreement with Table 5. Therefore, higher absolute error values are also associated with greater irradiance levels, although the reference condition to compute *G*, as introduced in [34], is the standard test condition (STC). Under STC, the irradiance is 1000 W·m^−^² and the cell temperature is 25 °C. Because the temperature effect on *I_mpp_* is not taken into account in [34], the absolute error tends to be greater as the temperature differs from 25 °C, despite the irradiance levels being close to 1000 W·m^−^². 

We compared the reliability of our method for the irradiance estimation with the reliability of those found in the literature and presented in the introduction. Our method estimates the solar irradiance with an nRMSE between 4.2% and 4.7%; from the state-of-the-art, it appears that some methods give a poorer value as 6%–8% for a short-circuit method [5], 6.4% and 6.8% for, respectively, a short-circuit and under-load methods [20] and even with an nRMSE equal to 15.1% for short-circuit method [29]; some other methods present an equivalent accuracy such as the method developed by Carrasco et al. [31] with a 0.87% and 6.67% nRMSE for clear sky and cloudy sky days, respectively. Better results are obtained by Laudani et al. [28] (nRMSE = 1.5–3.2%) and Moshksar and Ghanbari [33] with an average nRMSE of 1.08% (maximum 4.22%) for solar irradiance estimation.

Thus, it appears that our methodology, easy to implement and which does not require an interruption of production, allows the estimation of solar irradiance with an accuracy in the same order of magnitude to those found in the literature.

### 3.3. Plots of Errors and Measured and Calculated Parameters

**Figure 5:**
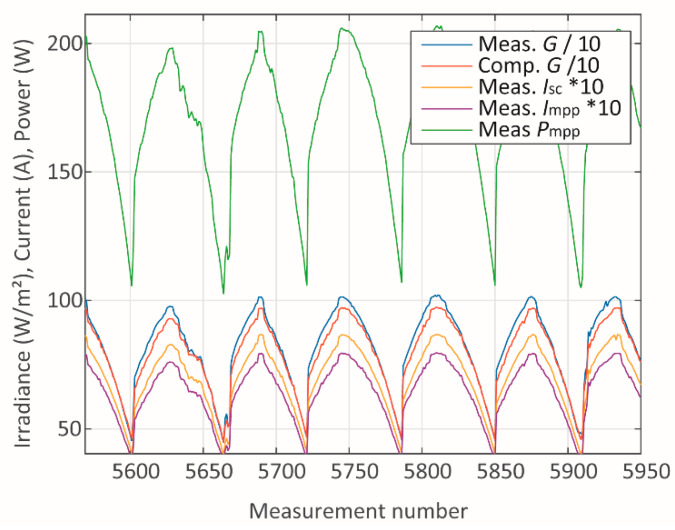
Irradiance, current, and power during clear-sky days.

**Figure 6:**
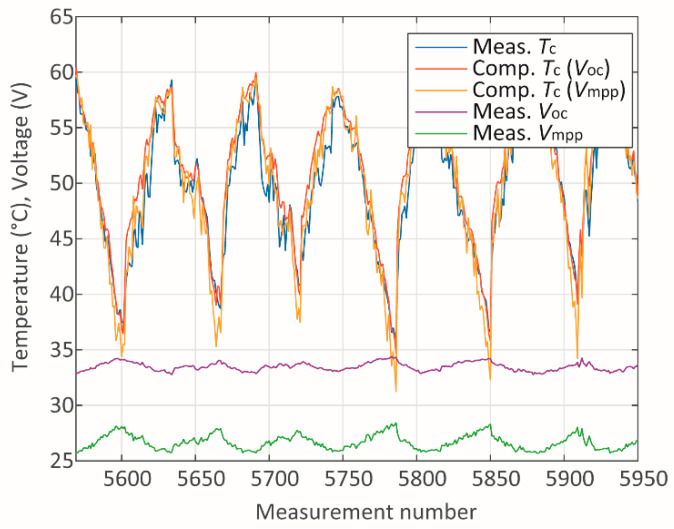
Temperature and voltage during clear-sky days.

**Figure 7:**
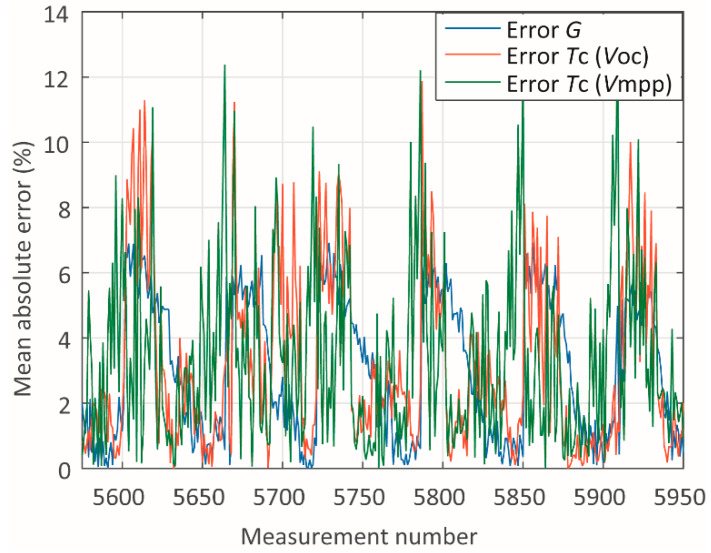
Absolute errors during clear-sky days.

**Figure 8:**
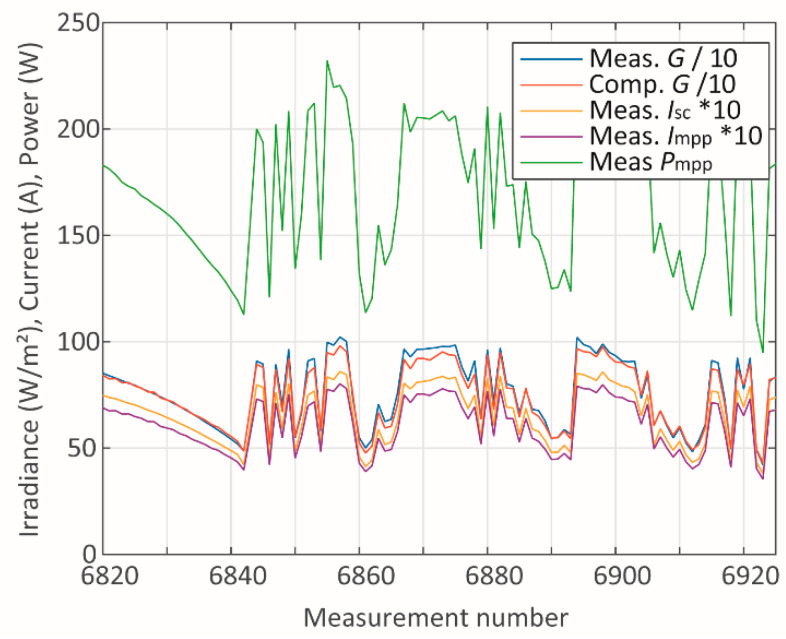
Irradiance, currents, and power during cloudy days.

**Figure 9:**
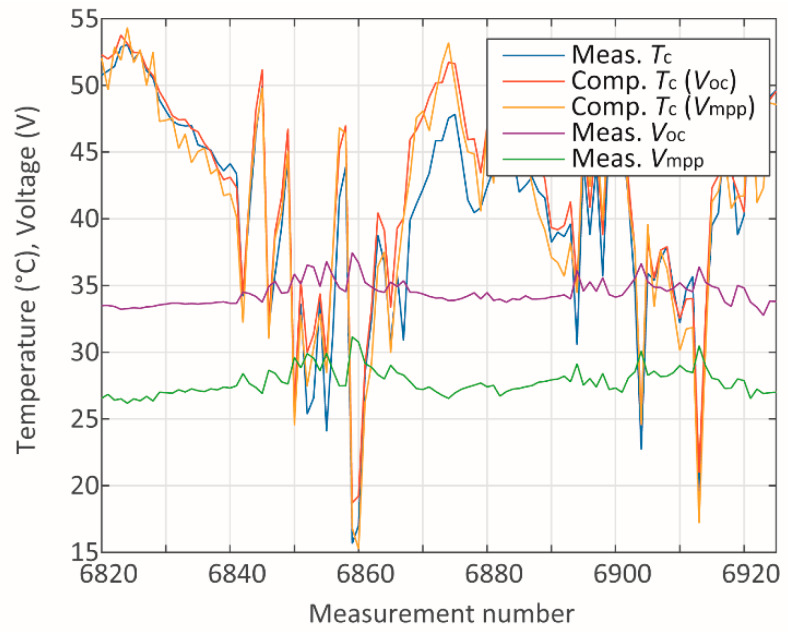
Temperature and voltage during cloudy days.

**Figure 10:**
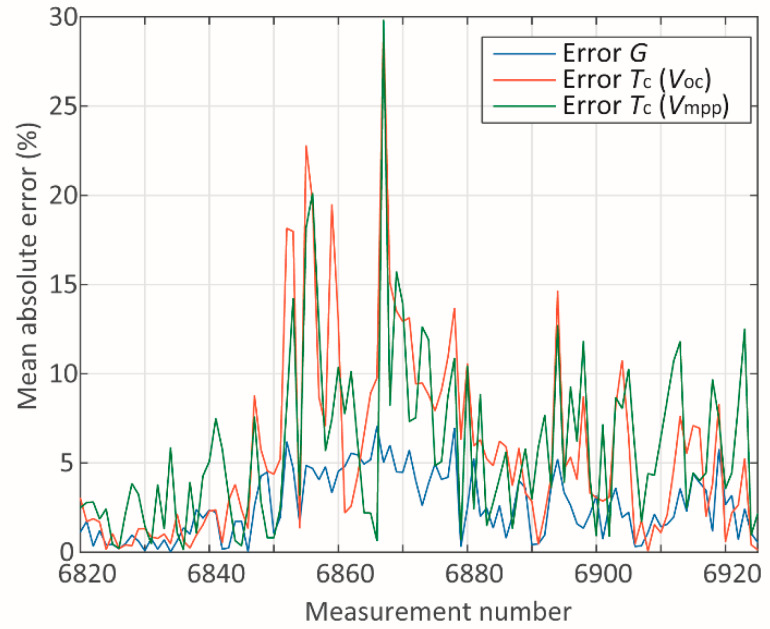
Absolute errors during cloudy days.

In addition to presenting the absolute mean errors for each parameter and module, a study of the sources of such errors has been carried out. The measured and computed data have been studied individually for each module in terms of the measured and computed data associated with it. Two cases referring to module A have been studied through the analysis of the plots in Figure 5, Figure 6, Figure 7, Figure 8, Figure 9 and Figure 10. One case refers to clear-sky days, where the irradiance (Figure 5) did not suffer interference from clouding; therefore, all parameters (Figure 5 and Figure 6) present relatively slow variation, and the error (Figure 7) is relatively low.

On the other hand, the second scenario regards days presenting steep variations on *G* (Figure 8), which reflects in abrupt variations of the other parameters (Figure 8 and Figure 9), resulting in relatively high peaks of absolute error (Figure 10). One reason for this is that steep variations of *G* introduce a transient condition, where an immediate response of voltages and currents is observed. As the temperature changes, the voltages vary accordingly, thus, applying the methods [34,35] to compute *T_c_* should provide reliable results. However, it should be recalled that the reference for the measurements are temperature sensors attached to the back of the modules. The response of these sensors presents a delay, which could increase the error when *T_c_* is computed in a strong transient condition. 

The irradiance level is used in both methods [34,35] to compute *T_c_* from *V_oc_* or *V_mpp_*, respectively. Although the effect of *G* itself on the voltages is instantaneous, its effect on *T_c_* (and therefore on *V_oc_* and *V_mpp_*, due to *T_c_*) takes some time to stabilize due to the thermal inertia of the module. As a result, under steep variations of *G*, the measurements of *G*, *T_c_*, and ambient temperature at a given instant would not seem coherent. Despite that, in this work, these values have been used to compute *T_c_*, even under transient condition. It should be noted that the use of a sensor as a reference for *T_c_* also contributes to increased errors under transitory condition, as the sensor has its associated thermal inertia. One possible approach to overcome such problems associated with steep variations of *G*, could be by means of monitoring *G* and the time step. If the ratio d*G*/dt is greater than a certain threshold value, then the methods should not be applied. 

Another source of greater levels of absolute error is the irradiance level under which *T_c_* has been computed. Regarding both methods [34,35], the modeling has been carried out under STC condition, whereas the adjustment (or tuning) has been performed based on the condition for NOCT. Therefore, as the modules operate under low irradiance levels, the absolute error increases. 

The plots in Figure 5 and Figure 8 illustrate, for some days, measured points under two different conditions, clear and cloudy sky. The nMAE and nRMSE have been computed for all the available measured data and are presented in Table 8. It shows, as expected, higher error levels under cloudy sky, owed to the transient effects already mentioned. The nRMSE values shown in Table 8 are within the values found by Carrasco et al. [31], which are 0.87% and 6.67% for clear sky and cloudy sky days, respectively.

**Table 8:** Normalized nMAE and nRMSE on computed parameters for the cases of clear sky (Figure 5) and cloudy sky (Figure 8).

	Clear Sky		Cloudy Sky	
	nMAE(%)	nRMSE (%)	nMAE(%)	nRMSE (%)
***G***	3.9	4.6	3.6	4.3
***T_c,Voc_***	4.7	5.8	4.5	5.8
***T_c,Vmpp_***	4.0	5.0	5.8	6.9

It is difficult to compare the results presented in Table 8 with those of Table 6 and Table 7; as noted previously, the Vco and Vmpp methods gave very close results in term of performances. A clear sky day and a cloudy day include solar irradiance levels in various ranges according to the time and the season, thus comparing the performances according to the irradiance level and the type of day is difficult to realize. Moreover, a more indepth study should be carried out about the classification in “cloudy” or “clear” days, thus providing a much more representative and reliable study by type of day.

## 4. Conclusions

Methods to calculate solar irradiance *G* and average cell temperature *T_c_* have been recently proposed, and their performance has been assessed in this work. Such methods have been applied to a large dataset, taken during almost 16 months, with I-V curves of 4 PV modules measured in 5-min intervals. The dataset thus contains measurements of electric parameters at the output of four PV modules, as well as the corresponding measured levels of *G* and *T_c_*. The irradiance has been computed by means of the application of [36], which uses *I_mpp_* measurements. In turn, *T_c_* has been calculated through the application of [35] (using *V_oc_* measurements) and [34], using *V_mpp_* values. It is worth mentioning that application of such methods to large PV plants, containing multiple strings of PV modules, could result in incoherent values of *G* and *T_c_* due to the electrical losses at the connection points, which influence the peak power and the associated voltage and current. This prospect will be studied in a further work, which is currently under development, and could lead to improvements in methods [34,35]. These methods are going to be used along with operation data records of a real PV plant. 

The nMAE and nRMSE for the calculation of *G* was between 3.5%–3.9% and 4.2%–4.7%, respectively, whereas the error on *T_c_* estimated via [39] was between 4.7%–8.1% for nMAE. Regarding *T_c_* estimated by method [34], error of 3.4%–8.2% (nMAE) and 4.3%–9.4% (nRMSE) has been observed. It was found that the application of both methods under unstable irradiance condition resulted in increased errors, due to the fact that the reference measurement for cell temperature is an RTD, which, provided its nature, presents a measurement lag under very dynamic conditions. 

The performances of our model were compared with that of the recent literature and it appears that the calculated nRMSE values are in good accordance with the values obtained by other authors.

A limitation of the method appeared for old modules, but a perspective of this work is to study in more detail and to take into account the aging effect.

Numerous technical works employ temperature sensors attached to the back of PV modules, allowing—in conjunction with a *G* meter—us to estimate the generated power under real operating conditions, at each instant. However, in this study, it has been demonstrated that the use of a temperature sensor as a reference for *T_c_*, under transient condition, is not a very effective alternative. First, PV cells as well as the most layers of the module need time to add to a new thermal equilibrium condition, due to changes in parameters, such as solar irradiance, wind speed, and temperature of the environment. In addition, the temperature sensor attached to the back of the PV module has a latency to adapt to this new balance condition. This resulted in relatively greater errors on computed *T_c_* levels when steep variations in *G* occurred. For this reason, we have proposed two methods [34,35]. Methods [34,35] do not present response latency, since the voltage is immediately affected by temperature and irradiance changes.

As a suggestion for future work, methods [34,35] could be evaluated for various PV technologies, such as c-Si, a-Si, CdTe, and CIS, under different weather conditions. The effects of factors such as module usage and tilt angle on the errors provided by the methods could also be assessed.

## Figures and Tables

**Table 1:** Parameters of each module at standard test conditions (STC), obtained from solar simulator tests.

	Module A	Module B	Module C	Module D
***V_oc_* (V)**	37.4	37.4	37.0	37.2
***I_sc_* (A)**	8.6	8.6	8.7	8.7
***V_mpp_* (V)**	30.5	30.5	30.1	30.3
***I_mpp_* (A)**	8.2	8.2	8.2	8.2
***P_mpp_* (W)**	249.4	248.7	247.0	248.2

**Table 2:** Datasheet information of Tenesol TE245-60M.

*V_oc_* (V)	*I_sc_* (A)	*V_mpp_* (V)	*I_mpp_* (A)	*P_mpp_* (W)	α (%/°C)	β (%/°C)	γ (%/°C)
37.4	8.7	29.8	8.3	247.3	0.0564	−0.348	−0.43

**Table 3:** Parameters of each module at nominal operating cell temperature (NOCT).

	Module A	Module B	Module C	Module D
***V_oc_* (V)**	34.3	34.4	34.2	34.5
***I_sc_* (A)**	7.1	7.0	7.0	7.0
***V_mpp_* (V)**	27.5	27.7	27.2	27.6
***I_mpp_* (A)**	6.5	6.4	6.5	6.5
***P_mpp_* (W)**	178.8	177.3	176.8	179.4

**Table 4:** Parameter values referring to methods [34,35].

	Module A	Module B	Module C	Module D
$\delta_{NOCT}$	0.0640	0.0498	0.0306	0.0195
ψ	0.0416	0.0164	0.0321	0.0085
τ	1.0434	1.2226	1.0796	1.0783

**Table 5:** Normalized nMAE and nRMSE on computed *G* per irradiance range, in %.

	50–300 W·m^−2^		300–600 W·m^−2^		600–1100 W·m^−2^		50–1100 W·m^−2^	
Module	nMAE	nRMSE	nMAE	nRMSE	nMAE	nRMSE	nMAE	nRMSE
**A**	3.8	4.2	3.7	4.4	4.0	4.6	3.9	4.7
**B**	3.5	3.9	3.6	4.1	3.5	4.1	3.5	4.2
**C**	3.4	3.7	2.8	3.4	4.0	4.4	3.7	4.4
**D**	3.3	3.6	2.9	3.5	3.9	4.4	3.7	4.4
